# Exploring the role of mitochondrial-associated and peripheral neuropathy genes in the pathogenesis of diabetic peripheral neuropathy

**DOI:** 10.1186/s12883-024-03589-0

**Published:** 2024-03-13

**Authors:** Ruojing Bai, Yuanyuan Luo

**Affiliations:** 1 Department of Geriatric Medicine, School of Clinical Medicine, Beijing Tsinghua Changgung Hospital, Tsinghua University, Beijing, China; 2 Department of Endocrinology and Metabolism, The First Affiliated Hospital of Zhengzhou University, Zhengzhou University, Zhengzhou, China

**Keywords:** Diabetic peripheral neuropathy, Protein–protein interaction, Mitochondrial dysfunction, Immune cell infiltration

## Abstract

**Background:**

Diabetic peripheral neuropathy (DPN) is a prevalent and serious complication of diabetes mellitus, impacting the nerves in the limbs and leading to symptoms like pain, numbness, and diminished function. While the exact molecular and immune mechanisms underlying DPN remain incompletely understood, recent findings indicate that mitochondrial dysfunction may play a role in the advancement of this diabetic condition.

**Methods:**

Two RNA transcriptome datasets (codes: GSE185011 and GSE95849), comprising samples from diabetic peripheral neuropathy (DPN) patients and healthy controls (HC), were retrieved from the Gene Expression Omnibus (GEO) database hosted by the National Center for Biotechnology Information (NCBI). Subsequently, differential expression analysis and gene set enrichment analysis were performed. Protein–protein interaction (PPI) networks were constructed to pinpoint key hub genes associated with DPN, with a specific emphasis on genes related to mitochondria and peripheral neuropathy disease (PND) that displayed differential expression. Additionally, the study estimated the levels of immune cell infiltration in both the HC and DPN samples. To validate the findings, quantitative polymerase chain reaction (qPCR) was employed to confirm the differential expression of selected genes in the DPN samples.

**Results:**

This research identifies four hub genes associated mitochondria or PN. Furthermore, the analysis revealed increased immune cell infiltration in DPN tissues, particularly notable for macrophages and T cells. Additionally, our investigation identified potential drug candidates capable of regulating the expression of the four hub genes. These findings were corroborated by qPCR results, reinforcing the credibility of our bioinformatics analysis.

**Conclusions:**

This study provides a comprehensive overview of the molecular and immunological characteristics of DPN, based on both bioinformatics and experimental methods.

**Supplementary Information:**

The online version contains supplementary material available at 10.1186/s12883-024-03589-0.

## Introduction

Diabetic peripheral neuropathy (DPN) stands as the most widespread complication associated with diabetes, representing the predominant form of neuropathy and a primary driver of disability, foot ulceration, and even amputation [1]. Furthermore, it has been reported that 20–30% of DPN patients experience neuropathic pain, often characterized by its chronic, severe, and challenging-to-treat nature [2–4]. Neuropathic caused by diabetic disease can be a debilitating condition that significantly impacts the quality of life for those affected. Although existing treatment methods provide relief from symptoms, they do not effectively impede the progression of the disease or reverse the damage inflicted on the nerves. The complexity of the disease arises from the interplay of various molecular mechanisms, pathways, and cellular components. Consequently, a thorough comprehension of the underlying molecular causes is essential for the development of precise therapeutic strategies.

At the same time, mitochondrial dysfunction and immune responses have been implicated in various complications of diabetes, but their specific role in DPN remains unclear. While some pathways like cellular responses, signaling, and autophagy have been explored, the complete array of biological processes contributing to DPN has yet to be fully elucidated.

The purpose of this study is to offer a comprehensive perspective on the intricate molecular alterations linked to the development of DPN. By pinpointing pivotal genes, pathways, and immune cell subtypes associated with DPN, this research aims to establish a foundation for the creation of precise therapeutic interventions that have the potential to enhance patient outcomes.

## Participants and methods

### Data source

Raw gene expression profile data of healthy control (HC) and DPN samples were retrieved from National Center for Biotechnology Information’s (NCBI) Gene Expression Omnibus (GEO) database (https://www.ncbi.nlm.nih.gov/geo), which is a public repository of functional genomics data. This study utilized GEO datasets GSE185011 and GSE95849. Table 1 provides details on the sample sizes and sequencing platforms for each cohort.

**Table 1:** Characteristics of the datasets

Set name	Total *n*	HC *n*	DPN *n*	Sequencing platform
GSE185011	10	5	5	GPL24676 (Illumina NovaSeq 6000)
GSE95849	12	6	6	GPL22448 (Phalanx Human lncRNA OneArray v1_mRNA)
Combined set (batch effect removed using Rank-In)	22	11	11	See above

### Statistical analysis

Statistical significance was determined utilizing a two-sided test. Statistical analyses were performed using R (v. 4.3.0, R Foundation for Statistical Computing, Vienna, Austria) and cystoscope (v. 3.1.10, Institute for Systems Biology, Seattle, W.A.) [5]. The Genome Reference Consortium Human Build 37 (GRCh37/hg19) was used as the reference genome.

### RNA sequencing data preprocessing

The duplicate ensembles or probes from each GEO dataset, which mapped to the same gene symbol, were averaged using the “avereps” function from the “limma” package [6]. Then, genes that had expression in less than 25% of samples of each dataset were removed. The TPM expression matrix of GSE185011 and the probe intensity of GSE95849 were log_2_-transformed and then standardized. Afterward, the two datasets were merged based on the same gene symbol to create a combined matrix. The Rank-In database (http://www.badd-cao.net/rank-in/index.html) [7] was used to identify and correct the batch effect.

### Differential expression analysis, functional enrichment analysis of GO and KEGG gene sets

The differentially expressed genes (DEGs) between normal tissues and disease tissues were identified using the moderated independent sample *t*-test from the “limma” package [8]. Genes with a |log_2_(fold change) [log_2_(FC)]| value of > 0.585 and a *p*-value < 0.05 were identified as differentially regulated.

Functional enrichment analysis aimed to provide functional interpretation for a list of genes that share a common characteristic, such as being differentially expressed [9]. After differentially expression analysis, statistical significance of the over-representation of significantly up-regulated [log_2_(FC) > 0.585 and *p* < 0.05] and down-regulated genes (log_2_(FC) < 0.585 and *p* < 0.05) between HC and DPN groups in Gene Ontology (GO) terms was determined using a hypergeometric distribution test, also known as over-representation anlysis (ORA) (hereafter referred to as ORA–GO). The GO database encompasses three main subcategories: Cellular Component (CC), Molecular Function (MF), and Biological Process (BP). The DEGs were also subjected to KEGG ORA analysis (hereafter referred to as ORA–KEGG). The top 30 (if available) most significant terms within each GO category and KEGG were ranked based on gene ratio and visualized as lollipop charts, employing the “ggplot2” package [10].

Furthermore, significant GO terms and KEGG pathways related to the mitochondrion selected from the most significantly enriched ones, further in-depth analysis was conducted. Bayesian network graphs of GO terms related to the mitochondrion were created using the “bngeneplot” function from the “CBNplot” package [11]. In these graphs, genes within these pathways were represented as nodes, and their expression levels were depicted as edges, providing a visual representation of their interplay and relationships. KEGG pathwaysrelated to the mitochondrion and shared by the two datasets were selected. Pathway mechanism diagrams (KEGG maps) of these pathways were depicted, and DEGs involved in this pathway were annotated on it using the “ggKEGG” package (Sato et al., 2023). GO–ORA and KEGG–ORA were performed using the “clusterProfiler” package [12]. A *p*-value of < 0.05 was considered statistically significant.

### Immune infiltration analysis

The gene probe intensity values in the combined dataset were used to quantify the amount of immune cell infiltration in each sample, employing the gene set variation analysis (GSVA) algorithm [13]. Marker genes associated with 24 immune cell types were obtained from the research by Şenbabaoğlu et al. [14]. The GSVA scores for the 24 immune cell with the above-mentioned gene sets were calculated using the “GSVAutils” package [15].

Comparison of each immune cell relative abundance between DPN and HC samples was performed by a two-sample Wilcoxon’s test provided by the “rstatix” package. Furthermore, correlations between expression levels of hub genes and immune cell abundances, and the association between immune cell abundances were assessed using Spearman’s rank correlation coefficient.

Correlation analyses were conducted to assess the relationships between the immunogenicity scores (IS) of 24 immune cells and the core genes. Spearman correlation analysis was employed for this purpose, and the *p*-values and Spearman correlation coefficients (r) were computed using the “Hmisc” package [16]. Significance was established for *p*-values less than 0.05.

### Identification and visualization of mitochondrion-associated and PND-associated DEGs

A comprehensive set of mitochondrion-associated genes was compiled by consulting multiple sources, including the MitoCarta 3.0 database (https://www.broadinstitute.org/mitocarta/mitocarta30-inventory-mammalian-mitochondrial-proteins-and-pathways), NCBI’s Genome database (https://www.ncbi.nlm.nih.gov/genome), and the MitoProteome Human Mitochondrial Protein Database (http://mitoproteome.org/). Concurrently, peripheral nerve disease (PND)-associated genes were retrieved from the Comparative Toxicogenomics Database (CTD, https://ctdbase.org/) and the DISEASES Database (https://diseases.jensenlab.org). The aggregated lists of mitochondrion-associated and PND-associated gene names are presented in Supplementary Table 1.

A comprehensive set of mitochondrion-associated genes was compiled by consulting multiple sources, including the MitoCarta 3.0 database (https://www.broadinstitute.org/mitocarta/mitocarta30-inventory-mammalian-mitochondrial-proteins-and-pathways), NCBI’s Genome database (https://www.ncbi.nlm.nih.gov/genome), and the MitoProteome Human Mitochondrial Protein Database (http://mitoproteome.org/). Concurrently, peripheral nerve disease (PND)-associated genes were retrieved from the Comparative Toxicogenomics Database (CTD, https://ctdbase.org/) and the DISEASES Database (https://diseases.jensenlab.org). The aggregated lists of mitochondrion-associated and PND-associated gene names are presented in Supplementary Table 1.

Subsequently, the mitochondrion-associated genes and PND-associated genes were respectively extracted from the differential analysis results of the two GEO datasets. To visually represent the significance and directionality of gene expression differences, volcano plots were generated for differentially expressed mitochondrion-associated genes and PND-associated genes using the “ggplot2” package [10]. In these plots, the x-axis represents the log_2_(fold change) while the y-axis represents the -log_10_(*p*-value). Shared differentially expressed mitochondrion-associated genes and PND-associated genes were identified by considering only those genes that consistently showed upregulation or downregulation across both GEO datasets. These selected genes were then chosen for further analysis.

For a comprehensive and visually intuitive representation of the overlapping genes, the “OmicCircos” package [17] was used to generate an omic circos diagram. This diagram visually displays the positions and expression levels of the shared differentially expressed mitochondrion-associated genes and PND-associated genes, mapping them onto the circular chromosome track.

### Establishment of PPI network and GO functional enrichment analysis

The Search Tool for the Retrieval of Interacting Genes/Proteins (STRING, v. 12.0) database (https://string-db.org/) [18] was used to establish the PPI network involving genes and proteins associated with mitochondria and PND. Additionally, the combined score for each predicted static PPI was calculated in this process. In this step, *homo sapiens* was selected as the organism for searching. The database is capable of predicting interactions among differentially expressed genes, considering both physical binding and regulatory interactions. It evaluates various aspects, including the strength of evidence, confidence, and molecular actions between the genes. To comprehensively explore the PPI data, the exploration parameters to encompass all available sources was configured, and the minimum required interaction score was set at a medium confidence level of 0.150.

To functionally characterize the PPI network, the STRING database was also used to perform GO functional enrichment analysis with an FDR threshold of 5%. Subsequently, the data representing the significantly enriched GO terms and the genes associated with these terms were visually presented through a heatmap, created using the “ggplot2” package [10].

### Identification of regulatory biomolecules

Regulatory molecules such as transcription factors (TFs) and microRNAs (miRNAs) are responsible for significant changes in transcription and expression outcomes. To anticipate miRNA-mRNA interactions, datasets were sourced from MiRcode (http://www.mircode.org/) and starBase (https://starbase.sysu.edu.cn/). Afterward, the prediction results were combined from both datasets through intersection to yield a more robust and consolidated set of miRNA-mRNA interactions. In addition, the datasets downloaded from the TransmiR v2.0 web tool (http://www.cuilab.cn/transmir) to predict TF–miRNA pairs. To focus on the most significant regulatory molecules, the interactions to include only those with a degree centrality exceeding 5 were filtered, encompassing TF-miRNA interactions and miRNA-gene pairs. Collectively, a regulatory network comprising TF-miRNA-mRNA interactions was constructed, involving genes associated with mitochondria and PND, in connection with OS and their respective upstream TFs and miRNAs. This network was constructed utilizing the “ggraph” package [19].

### Selection of hub genes

StringApp, an app integrated with Cytoscape, was employed to import PPI networks of DEGs associated with mitochondria and PND [20]. To further discover the hub genes in the complex PPI network, the CytoNCA plugin [21] in Cytoscape was employed. The CytoNCA app was employed to extract data using three distinct calculation methods: Betweenness, Closeness, and Degree. Genes that ranked within the top 30% for all three of these measures were identified as hub genes.

### Hallmark–GSEA for hub genes

In the combined dataset, the 11 DPN samples were stratified into high and low-expression groups, utilizing the median expression level of each hub gene as the threshold. Subsequently, single-gene GSEA was conducted using the Hallmark gene set, which comprises 50 well-defined biological states or processes applicable to a wide range of cellular responses [22] (referred to as GSEA–Hallmark). This method aimed to evaluate the impact of individual hub gene expression levels on the transcript expression within the tissues of DPN patients. Statistical significance was determined by an FDR-adjusted *p*-value for enriched functional terms or pathways, with values below 0.05 indicating statistical significance.

### Prediction of chemical drug prediction

Drugs with direct evidence for treating DPN patients were extracted from the table provided by the CTD database. Predicted drug candidates linked to at least one hub gene were identified as potential treatment options. A molecular network depicting the relationships between gene targets and drugs was then visualized using the “ggraph” package.

### RNA extraction and qPCR data analysis

A set of 5 pairs of PBMC samples from both normal and DN cases was procured from the clinical settings at The First Affiliated Hospital of Zhengzhou University. All participants willingly provided informed consent for their participation in the study. The study protocol received ethical approval from the ethics committee of The First Affiliated Hospital of Zhengzhou University. Total RNA was extracted from the 10 samples using the TRIzol reagent (Invitrogen, China), following the manufacturer’s instructions. Subsequently, cDNA synthesis was carried out through reverse transcription, utilizing the SureScript First-strand cDNA Synthesis kit (Servicebio, China). The qPCR assay was executed using the CFX Connect Thermal Cycler (Bio-Rad, USA). Relative quantification of mRNA was determined employing the 2^−ΔΔCT^ method. Details of all primer sequences are provided in Table 2.

**Table 2:** The primer sequences of key genes for qRT-PCR

Primer	Sequencing	
GAPDH	Forward	GGAGCGAGATCCCTCCAAAAT
	Reverse	GGCTGTTGTCATACTTCTCATGG
HSPA5	Forward	TGTTACAATCAAGGTCTATGAAGGTG
	Reverse	CAAAGGTGACTTCAATCTGTGG
LRRK2	Forward	ACGCAGCGAGCATTGTACCTT
	Reverse	GGCTTCATGGCATCAACTTCA
MRPL22	Forward	ACAGTTGGGTGCGTTATGGATAC
	Reverse	AGGCAGTTGTGGAGGATAAACAA
MARS (MARS1)	Forward	TCAGTGTAAAGTCTGCCGATCAT
	Reverse	CCCATTTGAGGTCTCGGGTTATG

To analyze the differential expression of the hub gene, pairwise independent *t*-tests were performed and *p* values were adjusted for multiple testing using FDR method by the “rstatix” package [23]. The visualization of these results was achieved through a bee swarm plot, generated using the “ggplot2” package [10] and the “ggbeeswarm” package [24].

## Results

### Batch effect removement

The 3D scatter plots in Supplementary Fig. 1 illustrate principal component analysis (PCA) result of the original and corrected combined dataset. In Supplementary Fig. 1A, it is apparent that there is a need for batch adjustment in the combined dataset, as batch effects are evident. However, after employing the Rank-In web tool for batch adjustment, as depicted in Supplementary Fig. 1B, there is no longer any discernible evidence of batch effects. Supplementary Fig. 1C further confirms that the samples are now primarily separated by condition and not influenced by batch effects, as observed in the initial Supplementary Fig. 1A.

### Identification of DEGs

Differential gene expression analysis between the DNP samples and HC tissues revealed 8637 and 1509 significant DEGs in GSE185011 and GSE95849 respectively (*p* < 0.05 and |log_2_(FC)|> 0.585). Among these DEGs, the first dataset showed 557 downregulated genes and 952 upregulated genes, and the second dataset exhibited 4534 downregulated genes and 4103 upregulated genes (Supplementary Table 2).

### Enriched GO Terms and KEGG Pathways

**Fig. 1:**
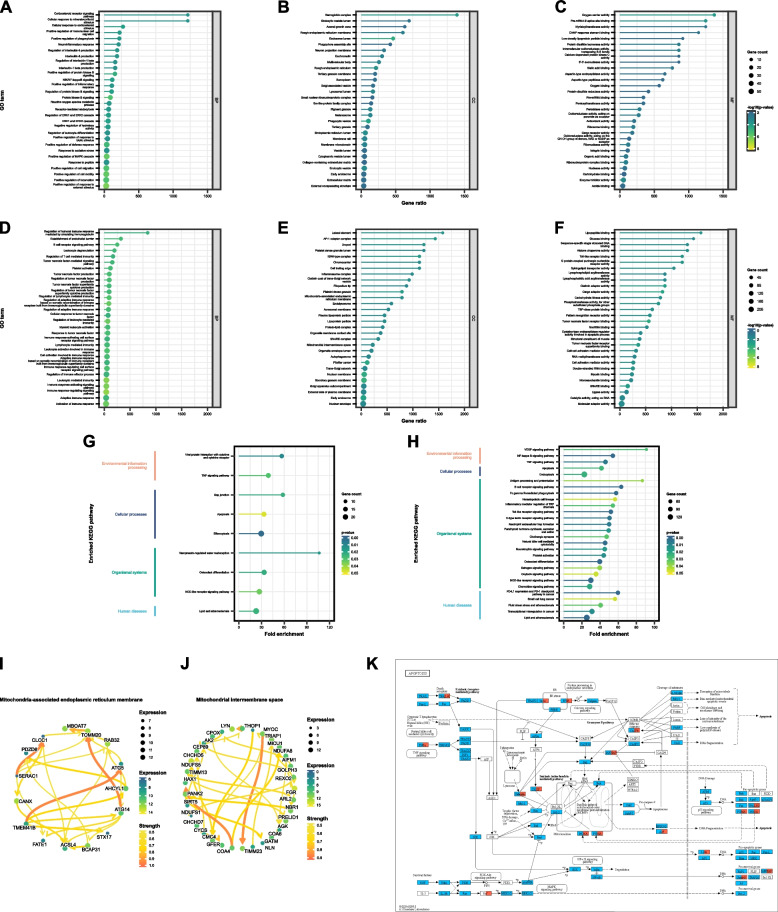
GO terms and KEGG pathways significantly altered in the DPN vs. HC comparison. Enriched GO terms and KEGG signaling pathways associated with dysregulated genes. Subsequently, Over-Representation Analysis (ORA) was conducted for Biological Process (BP), Cellular Component (CC), and Molecular Function (MF) subcategories. Figure 2A–F showcase the top 30 relevant GO terms within each ontology, featuring a *p*-value < 0.05 and ranked by gene ratio. Dot sizes correspond to the number of overlapping genes, while *p*-values are color-coded as per the legend’s scale. **A**–**C** Lollipop plots depict the top 30 GO terms for (**A**) BP, (**B**) CC, and (**C**) MF derived from DEGs in the GSE185011 dataset. **D**–**F** Lollipop plots display the top 30 GO terms for (**D**) BP, (**E**) CC, and (**F**) MF from DEGs in the GSE95849 dataset. **G**-**H** Lollipop plots illustrate the top 30 significantly enriched KEGG pathways from DEGs in GSE185011 and GSE95849 datasets, respectively. **I**–**L** Bayesian network (BN) plots demonstrate the relationships among the involved genes in significantly enriched GO terms and KEGG pathways related to the mitochondrion: (**I**) mitochondria-associated endoplasmic reticulum membrane (CC term) in the GSE185011 dataset; (**J**) mitochondrial intermembrane space (CC term) in the GSE185011 dataset; (**K**) KEGG raw map of the apoptosis pathway (hsa04210) indicating DEGs in GSE185011 (tomato red box) and GSE95849 datasets (sky blue box)

Figure 1A–F display the top 30 most enriched terms in three GO sections from the GSE185011 and GSE95849 datasets. ORA–GO results of GSE185011 dataset under BP and MF and subcategories revealed DPN’s association with immune responses and signaling pathways. Notably, terms like “immune response-regulating signaling pathway” and “tumor necrosis factor production” hint at the intricate link between immune responses and mitochondrial function, potentially mediated through reactive oxygen species (ROS) and other signaling molecules [25].

ORA–GO analysis on DEGs in GSE95849 dataset (Fig. 1E) identified pivotal CC related to mitochondrial structures in DPN patients, highlighting “mitochondria-associated endoplasmic reticulum membrane” and “mitochondrial intermembrane space.” These terms underscore the close interplay between the endoplasmic reticulum and mitochondria, crucial for calcium signaling, lipid metabolism, and apoptosis processes, and indicate mitochondrial dysfunction’s central role in DPN. The regulatory network of involved DEGs within each term is shown in Fig. 1I and J. Besides, results of BP ontology shed light on cellular responses and signaling pathways, marking a significant enrichment in terms related to the positive regulation of cell motility and migration, alongside inflammation and immune responses.

With regard to ORA–KEGG, a notable intersection between the datasets is the enrichment of the apoptosis pathway (hsa04210), directly tying back to mitochondrial dysfunction. This shared pathway, illustrated in the raw maps (Fig. 1K), emphasizes the interconnectedness of apoptosis with DPN, underscoring the role of mitochondrial health in cell survival and death mechanisms. Therefore, the importance of DEGs associated with mitochondria dysfunction and PND in the occurrence and development of DPN patients were further explored.

### Identification of hub mitochondrion-associated and PND-associated DEGs

The differential gene expression between DPN patients and healthy controls were illustrated utilizing volcano plots, with a keen focus on DEGs associated with mitochondria dysfunction and PND. This visualization strategy not only highlights the genes with significant expression changes but also underscores their statistical relevance, facilitating a rapid, intuitive understanding of key molecular players [26].

**Fig. 2:**
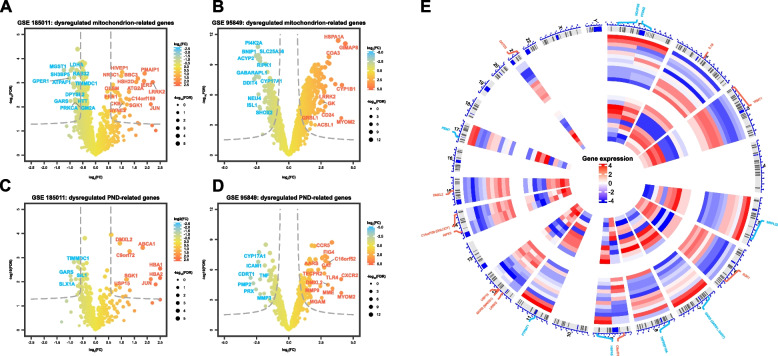
Dysregulation of mitochondrion-associated and mitochondrion-associated genes in individuals in the DPN group. Volcano plots showing DEGs in tissues from DPN patients vs. HC participants: **A** dysregulated mitochondrion-associated genes in GSE185011 dataset; **B** dysregulated PN-associated genes in GSE185011 dataset; **C** dysregulated mitochondrion-associated genes in GSE95849 dataset; **D** dysregulated PND-associated genes in GSE95849 dataset. The size of each data point is proportional to the -log_10_(FDR adjusted *p*-value), and log_2_(fold change) values are color-coded according to the provided scale. **E** Omic circos diagram displaying the position and expression levels of the shared differentially mitochondrion-associated and PND-associated genes mapping to their position on the chromosome circular track (the outer track). The two heatmap track display the expression level of the 19 shared differentially mitochondrion-associated genes and PND-associated genes in DPN patients compared to HC participants in GSE185011 (the first heatmap track) and GSE95849 (the second heatmap track) datasets, with heatmap color representing expression levels

The comparative analysis between the DPN patient group and controls, particularly within the GSE185011 and GSE95849 datasets, revealed a distinct expression pattern of mitochondrial and PND-associated genes. The GSE185011 dataset showed 70 downregulated and 35 upregulated mitochondrial-associated genes (Fig. 2A), with 16 downregulated and 39 upregulated PND-associated genes (Fig. 2B). Specifically, the GSE95849 dataset exhibited 308 downregulated and 494 upregulated mitochondrial-associated genes (Fig. 2C), alongside 117 downregulated and 41 upregulated PND-associated genes (Fig. 2D). This differential expression highlights the significant impact of mitochondrial dysfunction and peripheral nerve damage in the pathophysiology of DPN.

A critical step in our analysis was the intersection of DEGs from both datasets, revealing 19 genes associated with both mitochondrial function and PND. This subset of genes, including downregulated genes like GARS1 and HSPA5 and upregulated genes like DGLUCY and SGK1, provides a focused list of candidates for further investigation. These genes, implicated in processes ranging from mitochondrial biogenesis to inflammation, offer valuable insights into the complex interplay between mitochondrial dysfunction and peripheral nerve damage in DPN.

The research also extended to the chromosomal distribution of these key DEGs, with a notable presence on chromosomes such as 1, 9, 12, and 14 (Fig. 2E). This distribution pattern may suggest specific genomic regions that are particularly relevant to the pathogenesis of DPN, shedding light on the genetic underpinnings of this condition.

### Hub genes in the PPI network

**Fig. 3:**
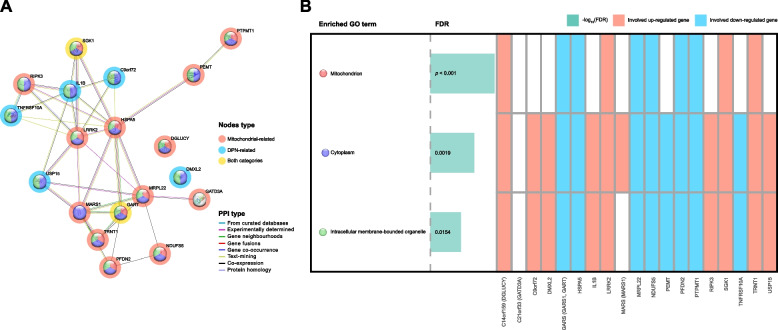
The PPI network of 19 shared DEGs associated with either mitochondrial dysfunction or PND and enriched GO terms. **A** This network contains a total of 19 proteins and 37 edges using STRING database (confidence cutoff of 0.150). The color of nodes represents the enriched GO terms they are associated with. Genes linked to mitochondria are encircled by a red halo, PND-associated genes by a blue halo, and genes relevant to both categories by a yellow halo. **B** Heatmap plot of enriched GO terms (FDR < 0.05) and involved proteins from the STRING database

**Fig. 4:**
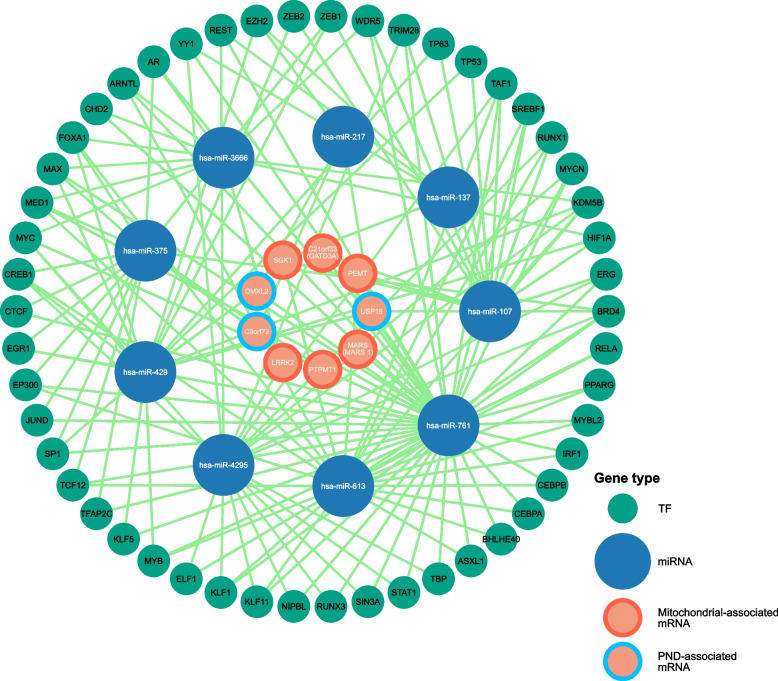
Gene regulatory (mRNA–miRNA–TF) network associated with the shared mitochondrial-associated and PND-associated genes

A PPI network (Figs. 3 and 4) was constructed using the STRING database to explore the interconnections among 19 shared DEGs associated with either mitochondrial dysfunction or PND in DPN patients versus healthy controls (Supplementary Table 3). Among the network’s nodes, HSPA5 emerges as a pivotal hub, connecting with 10 other genes, signifying its potential central role in DPN's molecular landscape. Following closely, LRRK2’s interactions with nine genes further highlight its importance. Other notable genes, such as GARS and MARS, with six interactions each, and IL1B and RIPK3, each with five, suggest their significant roles in DPN. The network also reveals genes like C21orf33 and C14orf159 with minimal connections, hinting at unique, potentially critical roles within the broader interaction map.

The GO functional enrichment analysis of the PPI network yielded significant insights, identifying key ontological terms such as mitochondrion, cytoplasm, and intracellular membrane-bounded organelle. These terms reflect the underlying biological themes connecting DPN with broader cellular functions and dysfunctions, particularly emphasizing the mitochondrial involvement.

Employing the CytoNCA plugin [21] within Cytoscape, our analysis pinpointed hub mRNAs based on degree centrality, betweenness centrality, and closeness centrality, offering a multidimensional view of node importance within the network (Supplementary Table 4). This approach consistently highlighted HSPA5, LRRK2, MRPL22, and MARS (MARS1) as hub genes, underscoring their potential as key players in DPN pathogenesis and their link to both mitochondrial dysfunction and PND.

### Transcriptional and post-transcriptional biomarkers

**Fig. 5:**
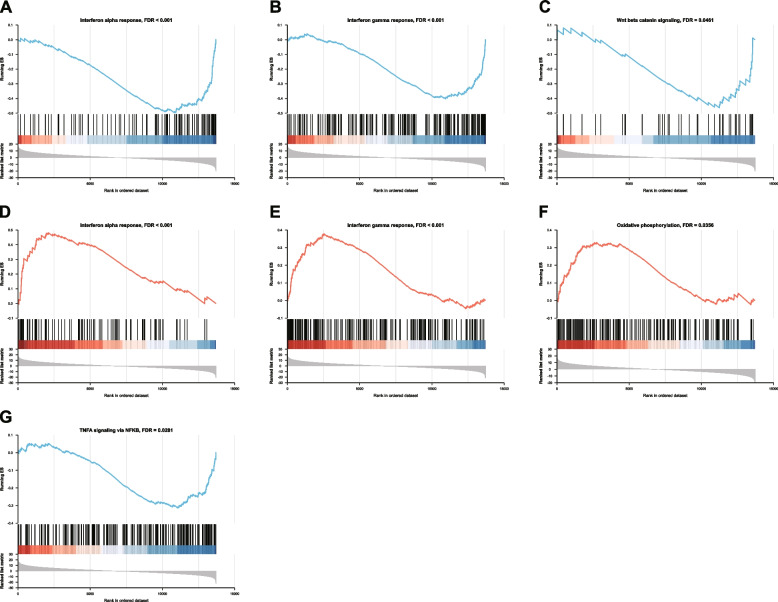
Most significantly enriched Hallmark gene sets related to the class distinction. HSPA5 exhibited significant negative correlations in **A** interferon alpha response and interferon gamma response, and **B** Wnt beta-catenin signaling. LRRK2 exhibited significant positive correlations in **C** interferon alpha response and **D** interferon gamma response. MRPL22 showed a significant positive correlation with **E** oxidative phosphorylation. MARS gene displayed a significant negative correlation with **F** TNFA signaling via NFKB. The score at the peak of the plot (the score furthest from 0) is the ES for the overall gene set. The middle portion of the plot shows where the members of the gene set appear in the ranked list of genes. The bottom portion of the plot shows the value of the ranking metric as the list of ranked genes decreases in value. The ranking metric measures an individual gene’s correlation between its expression and the related to the phenotypes (expression level group)

This study also established an intricate regulatory network (Fig. 5 and Supplementary Table 5) that illustrate the expression of shared DEGs associated with mitochondrial dysfunction and PND in DPN. By predicting miRNAs that regulate the 19 shared DEGs and subsequently identifying TFs associated with these miRNAs, we construct a comprehensive regulatory network. This network retains 9 key mRNAs, offering a window into the complex transcriptional and post-transcriptional landscape.

In this network, 45 miRNAs show potential regulatory relationships with the DEGs. Specific miRNAs, such as hsa-miR-107, hsa-miR-137, and hsa-miR-217, among others, emerged as critical regulators, associated with multiple TFs and mRNAs. These miRNAs represent key elements in the regulatory cascade, influencing the expression of genes pivotal to mitochondrial and PND-associated pathways, potentially driving the progression of mitochondrial dysfunctions and PND.

The regulatory influence extends to 29 TFs, intricately linked to the miRNAs and mRNAs, illustrating a complex web of gene expression regulation. These TFs, by interacting with the identified miRNAs, could modulate the expression patterns of the 19 genes, further elucidating the transcriptional landscape central to the pathogenesis of DPN.

### Enriched hallmark terms in single-gene GSEA

**Fig. 6:**
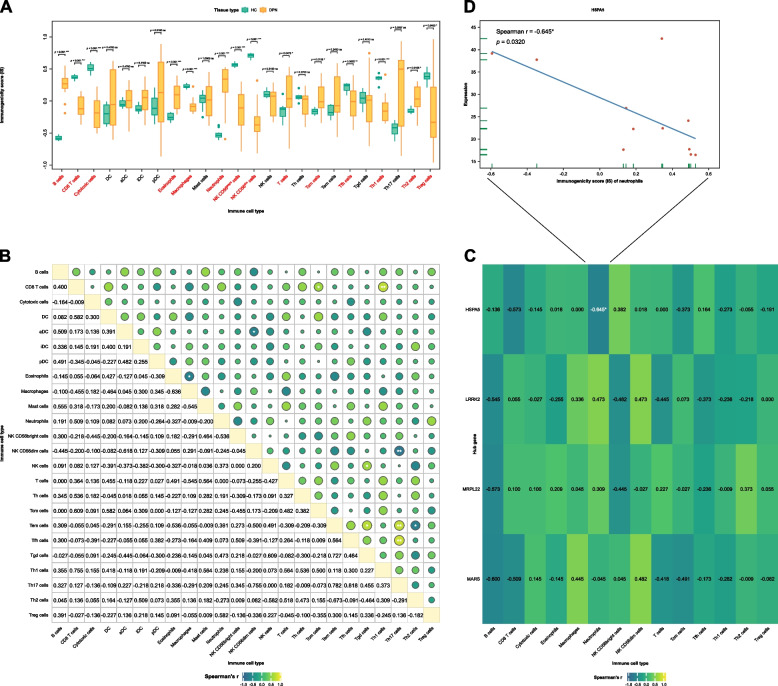
Immune microenvironment analysis. **A** Boxplot showing the differences in immunogenicity score (IS) between tissues from the DPN patients and HC participants. **B** Heatmap showing the association between the immunogenicity score (IS) of 24 immune cells in DPN patients. **C** Heatmap showing the association between the four hub genes and the immunogenicity score (IS) of 24 immune cells in DPN patients. **D** Scatter plot showing the association between the expression level of the HSPA5 gene and the IS of neutrophils in DPN patients

The results of single-gene GSEA (Fig. 6) revealed distinct patterns of association between the four hub genes (HSPA5, LRRK2, MRPL22, and MARS) and various hallmark gene sets. For instance, HSPA5 shows a significant inverse correlation with interferon responses and Wnt beta-catenin signaling, while LRRK2 exhibits a positive correlation with these interferon responses. MRPL22’s association with oxidative phosphorylation and MAR’'s negative correlation with TNFA signaling via NFKB highlight the diverse roles these genes play in cellular processes and stress responses, potentially impacting DPN pathology.

### Immune microenvironment analysis

A total of 24 immune cell subsets potentially contributing to the immune microenvironment of DPN were quantified using the GSVA method (Supplementary Table 6). Subsequently, the IS of 24 immune cell subsets in patients with DPN was compared with the HC group using the Wilcoxon’s test. The IS reflects the relative abundance and activation status of each immune cell subset in the peripheral blood. Figure 6A reveals significant variances in the IS of various immune cell subsets between DPN patients and the HC group. Notably, B cells, eosinophils, neutrophils, T cells, Central Memory T cells (Tcm), and T helper 2 cells (Th2) were found to be significantly reduced in the DPN group (*p* < 0.05). Conversely, the expression levels of CD8 T cells, cytotoxic cells, macrophages, nature killer (NK) CD56^bright^ cells, NK CD56^dim^ cells, Follicular helper T cells (Tfh), T helper (Th) 1 cells, and regulatory T cells (Treg) were significantly elevated in the DPN group (*p* < 0.05). No significant differences were observed for the remaining immune cell types.

Spearman’s correlation analysis provided further insights into the intricate relationships between these immune cell subsets (Fig. 6B). Notably, activated dendritic cells (aDC) showed significant positive correlations with B cells, cytotoxic cells, dendritic cells (DC), and T_cm_ cells (*p* < 0.05), while exhibiting significant negative correlations with CD8 T cells, NK CD56^bright^ cells, NK CD^56dim^ cells, Tfh cells, Th1 cells, and Th2 cells (*p* < 0.05). Other significant positive and negative correlations were observed among various immune cell subsets, highlighting a complex and balanced immune mechanism potentially involved in DPN pathology.

A noteworthy finding from our correlation analysis was the significant negative correlation between the IS of neutrophils and the expression level of the core gene HSPA5 (r = -0.645, *p* = 0.0320, Fig. 6C and D). This association suggests that an increased immunogenic presence of neutrophils correlates with decreased expression of HSPA5, a gene known for its role in cellular stress responses. This relationship adds a layer of complexity to our understanding of how immune cells and molecular signals interplay in the pathology of DPN.

### Candidate chemical drugs

**Fig. 7:**
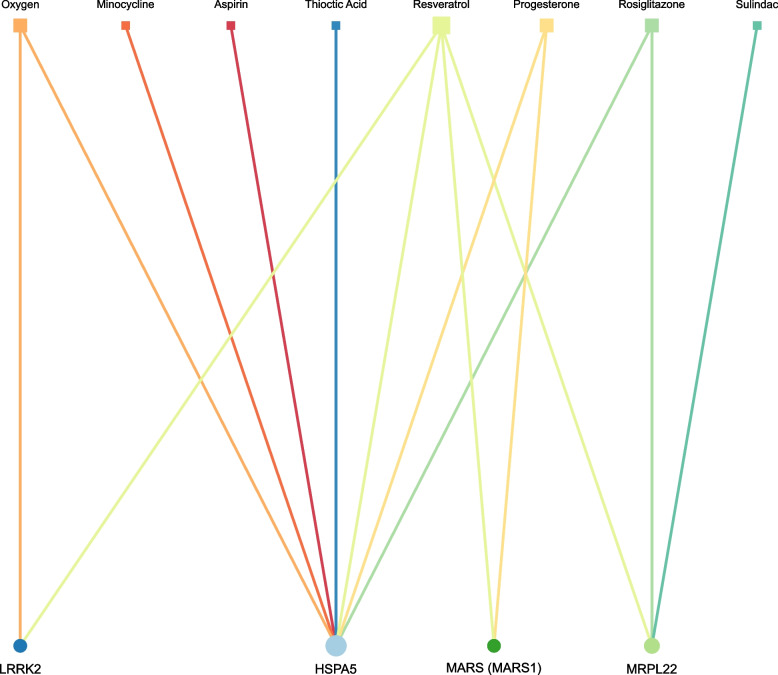
Protein-drug interaction network. Circle represent the hub dysregulated genes, while squares indicate the interacting drugs molecules. Node size is proportional to the degree (number of coincident edges)

Figure 7 illustrates the interactions between the four target hub genes and 8 corresponding chemical candidate drugs. Among these drugs, seven exhibited antagonistic relationships with HSPA5, while MRPL22 showed such relationships with three drugs. All these drugs have been found to be effective in treating DPN by interacting with gene targets, as supported by direct evidence. These drugs work through a variety of mechanisms. These drugs operate through various mechanisms, including reducing inflammation and alleviating pain (e.g., aspirin and sulindac), inhibiting bacterial infections (e.g., minocycline), improving hypoxic conditions (e.g., oxygen), promoting nerve repair and regeneration (e.g., progesterone), exerting antioxidant effects (e.g., resveratrol and thioctic acid), and lowering blood sugar levels (e.g., rosiglitazone), etc.

### Biological experiments

**Fig. 8:**
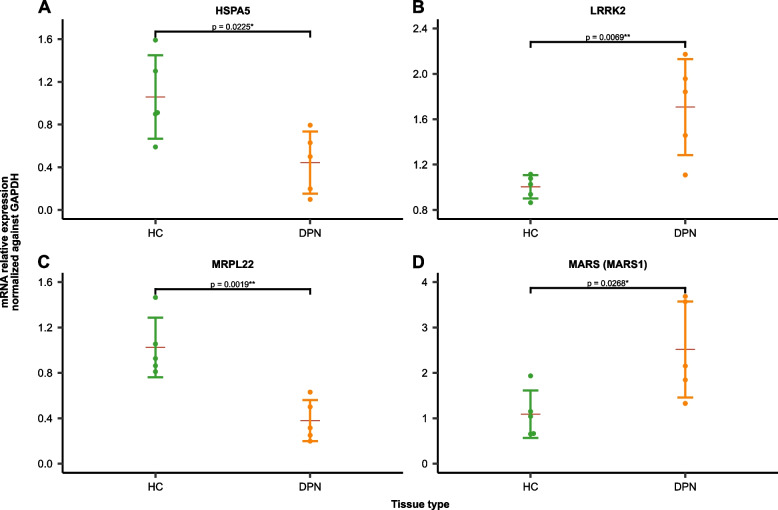
Dysregulated expression of four hub genes in individuals with DPN. The relative fold difference in **A** HSPA5, **B** LRRK2 **C** MRPL22, and **D** MARS (MARS1) mRNA expression levels in PBMCs from healthy control subjects (*n* = 5) and DPN patients (*n* = 5). The error bar shows average of five individuals ± SD. *p < 0.05, **p < 0.01 compared with HC participants, calculated by independent sample- *t* test

Four DE-GMRGs were selected for quantitative reverse transcriptase PCR (qRT-PCR) on clinical samples. The results of the study indicated that the expression levels of HSPA5 and MRPL22 were significantly higher in normal PBMC samples than in DN PBMC samples, while the LRRK2 and MARS (MARS1) were significantly higher in DN PBMC samples (Fig. 8).

## Discussion

The gene functional enrichment analyses conducted in our study, utilizing ORA–GO and ORA–KEGG, have delved into the intricate molecular landscape of DPN, revealing a diverse array of biological processes and pathways that distinguish DPN patients from healthy individuals. Our findings highlight the involvement of mitochondrial-associated membranes (MAM), intermembrane space (IMS), and a spectrum of cellular responses, signaling pathways, inflammation, immune modulation, autophagy, mitophagy, along with key aspects of energy metabolism, detoxification, and cellular homeostasis. This broad spectrum of dysregulated processes and pathways underscores the multifaceted nature of DPN pathogenesis, pointing to a confluence of multiple mechanisms and contributing factors.

Mitochondrial dysfunction has emerged as a pivotal factor contributing to the complexity of diabetic complications. This dysfunction, marked by oxidative stress and inflammatory responses, underscores the multifactorial nature of Type 2 Diabetes Mellitus (T2DM), where factors such as adipokines, insulin resistance, and beta-cell apoptosis interplay, exacerbating the disease’s progression [27]. The intricate relationship between these pathophysiological pathways and mitochondrial health highlights the necessity of addressing mitochondrial dysfunction in therapeutic strategies for DPN. Furthermore, the roles of autophagy and mitophagy in counteracting cellular damage induced by oxidative stress gain significance in the diabetic milieu. These protective mechanisms, by facilitating the removal of damaged mitochondria, offer a buffer against the deleterious effects of high glucose levels characteristic of T2DM, thus providing a potential therapeutic avenue to mitigate mitochondrial dysfunction and its implications in DPN [28].

The complexity of DPN ‘s pathophysiology is further compounded by the involvement of inflammation and immune responses, whose roles, albeit crucial, remain to be fully elucidated. The variance in these responses based on the disease's type and stage points to the nuanced nature of DPN and necessitates a deeper understanding of these mechanisms for effective management [29, 30]. Given the interconnection between mitochondrial dysfunction and immune responses in the context of DPN, exploring the expression of mitochondrial-related genes and DPN-associated genes becomes paramount.

The exploration of mitochondrial dysfunction in DPN patients through bioinformatics analysis sheds light on its potential to disrupt various gene ontologies and pathways. This disruption extends to nucleotide biosynthesis, fatty acid and ethanol metabolism, and drug metabolism, suggesting a comprehensive impact on cellular metabolism. The identification of such extensive metabolic alterations offers valuable insights into the metabolic dysregulation associated with DPN, reinforcing the importance of mitochondrial health in the disease’s pathology.

The PPI network analysis of mitochondrion-associated and PND-associated DEGs unveiled several notable interactions, such as between C21orf33 and MRPL22, and between C9orf72 and IL1B, providing a glimpse into the complex molecular interplay underlying DPN. The interaction between C21orf33, a mitochondrial membrane protein involved in mitochondrial biogenesis and morphology, and MRPL22, a mitochondrial ribosomal protein, suggests a coordinated role in maintaining mitochondrial function, which could be crucial in the development of peripheral nerve damage. Similarly, the link between C9orf72, associated with neurodegenerative diseases and neuronal apoptosis, and IL1B, a pro-inflammatory cytokine, underscores the potential interplay between neurodegeneration and inflammation in PND pathogenesis.

Furthermore, the connections between genes like GARS and LRRK2, both implicated in neurodegeneration, hint at their possible collective impact on mitochondrial function and nerve damage. These interactions, revealed through our PPI network and subsequent enrichment analysis, not only establish meaningful connections between mitochondrial function and PND but also lay the groundwork for deeper investigations into these genes’ roles in DPN’s development.

Moreover, the enriched ontological features within the PPI network, such as those associated with the mitochondrion, cytoplasm, and intracellular membrane-bounded organelles, suggest these cellular components' pivotal roles in DPN's pathogenesis. The involvement of these components highlights the intricate cellular dynamics at play in DPN, offering a more nuanced understanding of the disease's molecular underpinnings.

The construction of the TF–miRNA–mRNA network in our study provides a comprehensive view of the regulatory mechanisms that may influence the expression of mitochondrial-associated and PND-associated DEGs in DPN. This network underscores the intricate role of miRNAs in modulating gene expression, highlighting their capacity to fine-tune downstream signaling cascades and orchestrate transcription factor networks. For instance, the ability of hsa-miR-107 to suppress TAF1 and TP53 expression points to its influence on cell cycle regulation and apoptosis pathways. Similarly, hsa-miR-217's role in downregulating EZH2 expression may impact chromatin restructuring and gene expression regulation, while hsa-miR-375's targeting of CEBPA and CEBPB could affect metabolic and inflammatory pathways.

These miRNAs extend their regulatory impact beyond transcription factors, directly targeting critical mRNAs involved in DPN's pathogenesis. For example, hsa-miR-107's inhibition of USP15 and PEMT suggests its role in protein degradation and phospholipid metabolism, respectively. In contrast, hsa-miR-4295's suppression of LRRK2 expression, a gene implicated in neuronal function and neurodegeneration, highlights the potential of miRNAs in modulating key pathological processes in DPN. Additionally, hsa-miR-613's regulation of PPARG and RELA expression indicates its involvement in lipid metabolism and inflammatory responses, further illustrating the complex interplay between miRNAs, TFs, and mRNAs in the disease mechanism.

The identification of hub genes within the PPI network, such as HSPA5, LRRK2, MRPL22, and MARS1, emphasizes their significant roles in DPN's molecular landscape. These genes, associated with mitochondrial function or the peripheral nervous system, present differential expression patterns between DPN patients and healthy controls, suggesting their potential involvement in disease progression. The downregulation of HSPA5 and MRPL22, alongside the upregulation of LRRK2 and MARS1, provides a molecular signature that may underlie DPN’s pathophysiological changes. Each of these hub genes contributes uniquely to cellular processes, from stress response and protein folding (HSPA5) to mitochondrial dynamics (LRRK2), protein synthesis (MRPL22), and signal transduction (MARS1), painting a multifaceted picture of DPN's molecular drivers.

The single-gene GSEA further enriches our understanding of DPN by highlighting significant correlations between hub genes and various signaling pathways. Notably, the HSPA5 gene’s negative correlations with interferon alpha and gamma responses, alongside its inverse relationship with Wnt beta-catenin signaling, point to its potential roles in modulating anti-inflammatory responses, cell proliferation, and anti-oxidative stress mechanisms in DPN. Conversely, the positive correlations of the LRRK2 gene with interferon responses underscore its possible involvement in immune responses and neurodegenerative processes characteristic of DPN. The MRPL22 gene’s positive association with oxidative phosphorylation suggests its influence on mitochondrial function and energy metabolism, whereas the MARS gene's negative correlation with TNFA signaling via NFKB highlights its potential inhibitory effect on inflammatory cytokine production and action in DPN.

Our immune microenvironment analysis provides a detailed landscape of the immune cell dynamics in DPN, revealing significant differences in the expression levels of various immune cell subsets between DPN patients and healthy controls. The observed Th1-dominated immune response in the DPN group, characterized by increased expression of CD8 T cells, cytotoxic cells, macrophages, NK cells, Tfh cells, and Th1 cells, suggests a shift towards a pro-inflammatory state. This shift is further supported by the positive correlations between Th1 cells and other immune cell types, indicating a coordinated immune response potentially directed against self-antigens or infectious pathogens. Conversely, the negative correlations between Th1 cells and B cells, eosinophils, and neutrophils may reflect a transition from a Th2-dominated to a Th1-dominated immune response in DPN, implicating Th1 cells in cell-mediated immunity and the elimination of intracellular pathogens.

The complex interplay among the 24 immune cell types, as revealed by the significant correlations in their IS, underscores the intricate immune mechanisms involved in DPN's pathogenesis. These findings open new avenues for research into the roles of immune cells and hub genes in DPN and provide potential targets for diagnosis and treatment.

Lastly, the identification of eight potential drug candidates through gene-drug network analysis offers promising therapeutic avenues for DPN treatment. These drugs, with mechanisms ranging from reducing inflammation and alleviating pain to promoting nerve repair and regeneration, underscore the importance of a comprehensive, mechanism-based approach to DPN treatment.

Through exploring the molecular characteristics and immune microenvironment of DPN, this research is aimed at providing new insights for the diagnosis and treatment of DPN. Nevertheless, it is important to acknowledge certain limitations associated with this study that require consideration. Firstly, it’s important to note that the sample size in this study is relatively small, which may limit the generalizability of the findings to the broader DPN patient population. Consequently, further validation through larger-scale studies is warranted. Secondly, the data utilized in this study is sourced from public databases, introducing the possibility of biases and missing information. To enhance the robustness of the findings, there is a need to refine and optimize the analytical methods employed. Thirdly, it’s worth highlighting that this study exclusively explores a subset of pathways and genes associated with DPN, providing a partial perspective on its development. As such, the conclusions of this study should be complemented and refined through more extensive mechanistic research. Future investigations should aim to leverage higher-quality and more diverse data resources to address these limitations and offer a more comprehensive understanding of the pathological mechanisms underlying DPN.

## Conclusion

In conclusion, this study provides a comprehensive investigation into the molecular and immunological aspects of PPN using a multi-modal approach. It has successfully identified four hub genes—HSPA5, LRRK2, MRPL22, and MARS1—that exhibit significant associations with DPN pathology. These findings were not only established through bioinformatics methods but also validated using RT-qPCR experiments. Furthermore, the study delved into the regulatory roles of miRNAs on these hub genes and their associated pathways, shedding light on alterations in the immune microenvironment within DPN patients. An additional gene-drug network analysis revealed potential therapeutic options for DPN. Overall, these results provide valuable insights into potential mechanisms and therapeutic targets for DPN. To comprehensively elucidate the complex causal relationships contributing to DPN’s pathogenesis, further research is imperative.

### Supplementary Information

**Supplementary Material 1.**

**Supplementary Material 2.**

**Supplementary Material 3.**

**Supplementary Material 4.**

**Supplementary Material 5.**

**Supplementary Material 6.**

**Supplementary Material 7.**

## Data Availability

No datasets were generated or analysed during the current study.

## Abbreviations

ATP: Adenosine triphosphate
BP: Biological Process
CC: Cellular Component
CTD: Comparative Toxicogenomics Database
DEGs: Differentially expressed genes
DPN: Diabetic peripheral neuropathy
FC: Fold change
FDR: False Discovery Rate
GEO: Gene Expression Omnibus
GO: Gene Ontology
GSEA: Gene set enrichment analyses
GSVA: Gene set variation analysis
HC: Healthy control
IMS: Intermembrane space
IS: Immunogenicity scores
KEGG: Kyoto Encyclopedia of Genes and Genomes
MAM: Mitochondria-associated membranes
MF: Molecular Function
NCBI: National Center for Biotechnology Information
ORA: Over-representation analysis
PND: Peripheral nerve disease
PPI: Protein–protein interaction
STRING: Search Tool for the Retrieval of Interacting Genes/Proteins
