# Supplementary material for: Exploring the role of mitochondrial-associated and peripheral neuropathy genes in the pathogenesis of diabetic peripheral neuropathy
Source: BMC Neurol. 2024 Mar 13;24:95. doi: 10.1186/s12883-024-03589-0 (PMC10936109; doi:10.1186/s12883-024-03589-0)
Supplement: Supplementary file 7 — Supplementary Material 7. [file 12883_2024_3589_MOESM7_ESM.docx]

**Figure S1.** Principal component analysis (PCA) of the combined dataset. **(A)** PCA plot of gene expression level before removing batch effect in different datasets. PCA plot of gene expression level after removing batch effect in **(B)** different datasets and **(C)** samples.
